# Models of Classroom Assessment for Course-Based Research Experiences

**DOI:** 10.3389/feduc.2023.1279921

**Published:** 2023-11-27

**Authors:** David I. Hanauer, Tong Zhang, Mark J. Graham, Sandra D. Adams, Yesmi Patricia Ahumada-Santos, Richard M. Alvey, Mauricio S. Antunes, Mary A. Ayuk, María Elena Báez-Flores, Christa T. Bancroft, Tonya C. Bates, Meghan J. Bechman, Elizabeth Behr, Andrea R. Beyer, Rebecca L. Bortz, Dane M. Bowder, Laura A. Briggs, Victoria Brown-Kennerly, Michael A. Buckholt, Sharon K. Bullock, Kristen A. Butela, Christine A. Byrum, Steven M. Caruso, Catherine P. Chia, Rebecca A. Chong, Hui-Min Chung, Kari L. Clase, Sean T. Coleman, D. Parks Collins, Stephanie B. Conant, Brett M. Condon, Pamela L. Connerly, Bernadette J. Connors, Jennifer E. Cook-Easterwood, Katie E. Crump, Tom D’Elia, Megan K. Dennis, Linda C. DeVeaux, Lautaro Diacovich, Iain Duffy, Nicholas P. Edgington, Dustin C. Edwards, Tenny O.G. Egwuatu, Elvira R. Eivazova, Patricia C. Fallest-Strobl, Christy L. Fillman, Ann M. Findley, Emily Fisher, Matthew R. Fisher, Marie P. Fogarty, Amanda C. Freise, Victoria J. Frost, Maria D. Gainey, Amaya M. Garcia Costas, Atenea A. Garza, Hannah E. Gavin, Raffaella Ghittoni, Bryan Gibb, Urszula P. Golebiewska, Anna S. Grinath, Susan M. R Gurney, Rebekah F. Hare, Steven G. Heninger, John M. Hinz, Lee E. Hughes, Pradeepa Jayachandran, Kristen C. Johnson, Allison A. Johnson, Michelle Kanther, Margaret Kenna, Bridgette L. Kirkpatrick, Karen K. Klyczek, Kathryn P. Kohl, Michael Kuchka, Amber J. LaPeruta, Julia Y. Lee-Soety, Lynn O. Lewis, Heather M. Lindberg, Jaclyn A. Madden, Sergei A. Markov, Matthew D. Mastropaolo, Vinayak Mathur, Sean P. McClory, Evan C. Merkhofer, Julie A. Merkle, Scott F. Michael, Jon C. Mitchell, Sally D. Molloy, Denise L. Monti, María Alejandra Mussi, Holly Nance, Fernando E. Nieto-Fernandez, Jillian C. Nissen, Imade Y. Nsa, Mary G. O’Donnell, Shallee T. Page, Andrea Panagakis, Jesús Ricardo Parra-Unda, Tara A. Pelletier, Tiara G. Perez Morales, Nick T. Peters, Vipaporn Phuntumart, Richard S. Pollenz, Mary L. Preuss, David P. Puthoff, Muideen K. Raifu, Nathan S. Reyna, Claire A. Rinehart, Jessica M. Rocheleau, Ombeline Rossier, Adam D. Rudner, Elizabeth E. Rueschhoff, Amy Ryan, Sanghamitra Saha, Christopher D. Shaffer, Mary Ann V. Smith, Amy B. Sprenkle, Christy L. Strong, C. Nicole Sunnen, Brian P. Tarbox, Louise Temple, Kara R. Thoemke, Michael A. Thomas, Deborah M. Tobiason, Sara S. Tolsma, Julie Torruellas Garcia, Megan S. Valentine, Edwin Vazquez, Robert E. Ward, Catherine M. Ward, Vassie C. Ware, Marcie H. Warner, Jacqueline M. Washington, Daniel E. Westholm, Keith A. Wheaton, Beth M. Wilkes, Elizabeth C. Williams, William H. Biederman, Steven G. Cresawn, Danielle M. Heller, Deborah Jacobs-Sera, Graham F Hatfull, David J. Asai, Viknesh Sivanathan

**Affiliations:** 1 Department of English, Indiana University of Pennsylvania, 1011 South Drive, Indiana, PA 15705, USA; 2 Department of English, Duke Kunshan University, Duke Avenue, Kunshan, Jiangsu 215316, China; 3 STEM Program Evaluation and Research Lab (STEM-PERL), Department of Ecology and Evolutionary Biology, Yale University, 165 Prospect St, New Haven, CT 06511, USA; 4 Biology, Montclair State University, 1 Normal Avenue, Montclair, NJ 07043, USA; 5 Unidad de Investigaciones en Salud Pública. Facultad de Ciencias Químico Biológicas, Universidad Autónoma de Sinaloa, Ciudad Universitaria, CP 80010, Culiacán, Sinaloa 80010, USA; 6 Biology, Illinois Wesleyan University, 1312 Park St., Bloomington, IL 61701, USA; 7 Department of Biological Sciences, University of North Texas, 1155 Union Circle, Denton, TX 76203, USA; 8 Department of Biology, Howard University, 415 College St. NW, Washington, DC 20059-0001, USA; 9 Biological Sciences, University of Southern California, 3651 Trousdale Parkway, Los Angeles, CA 90089, USA; 10 Biological Sciences, The University of North Carolina at Charlotte, 9201 University City Blvd., Charlotte, NC 28223, USA; 11 Department of Biological Sciences, University of Pittsburgh, 4200 Fifth Avenue, Pittsburgh, PA 15260, USA; 12 Biology Department, Madison Area Technical College, 1701 Wright Street, Madison, WI 53704, USA; 13 Department of Biology, Virginia State University, 1 Hayden Drive, Petersburg, VA 23806, USA; 14 Department of Biology, Doane University, 1014 Boswell Ave., Crete, NE 68333, USA; 15 Biology, Truckee Meadows Community College, 7000 Dandini Blvd, Reno, NV 89512, USA; 16 Natural Sciences and Mathematics, Webster University, 407 E Lockwood Ave, St. Louis, MO 63119, USA; 17 Biology and Biotechnology, Worcester Polytechnic Institute, 100 Institute Road, Worcester, MA 01609, USA; 18 Biological Sciences, University of North Carolina at Charlotte, 9201 University City Blvd, Charlotte, NC 28223-0001, USA; 19 Department of Biology, College of Charleston, 66 George Street, Charleston, SC 29424, USA; 20 Department of Biological Sciences, University of Maryland, Baltimore County, 1000 Hilltop Circle, Baltimore, MD 21250, USA; 21 School of Biological Sciences, University of Nebraska-Lincoln, Lincoln, NE 68588, USA; 22 School of Life Sciences, University of Hawaii at Manoa, 3190 Maile Way, St. John 101, Honolulu, HI 96822, USA; 23 Biology, University of West Florida, 11000 University Parkway, Pensacola, FL 32514, USA; 24 Agricultural and Biological Engineering, Purdue University, 225 S. University Street, West Lafayette, IN 47907-2093, USA; 25 Biology, Wartburg College, 100 Wartburg BLVD, Waverly, IA 50677-1013, USA; 26 Natural Sciences, Mitchell Community College, 500 West Broad Street, Statesville, NC 28677, USA; 27 Department of Biology, University of Detroit Mercy, 4001 W. McNichols, Detroit, MI 48201, USA; 28 Biomedical Sciences, Rocky Vista University - Montana College of Osteopathic Medicine, 4130 Rocky Vista Way, Billings, MT 59106, USA; 29 School of Natural Sciences, Indiana University Southeast, 4201 Grant Line Rd., New Albany, IN 47150, USA; 30 Science Department, Dominican University New York, 470 Western Highway, Orangeburg, NY 10962, USA; 31 Biology, Queens University of Charlotte, 1900 Selwyn Ave., Charlotte, NC 28274, USA; 32 Department of Biologcal Sciences, Nova Southeastern University, 3301 College Ave., Fort Lauderdale, FL 33314, USA; 33 Department of Biology, Indian River State College, 3209 Virginia Ave, Fort Pierce, FL 34981-5541, USA; 34 Biology, Marist College, 3399 North Road, Poughkeepsie, NY 12601, USA; 35 Biology Department, New Mexico Institute of Mining and Technology, 801 Leroy Pl., Socorro, NM 87801, USA; 36 Departamento de Microbiología Básica, Facultad de Ciencias Bioquímicas y Farmacéuticas-UNR, Suipacha 531, Rosario, Santa Fe 2000, Argentina; 37 Department of Natural Science, Saint Leo University, 33701 County Road 52, Saint Leo, FL 33574, USA; 38 Dept. of Biology, Southern Connecticut State University, 501 Crescent St., New Haven, CT 06515, USA; 39 Department of Biological Sciences, Tarleton State University, 1333 W. Washington, Stephenville, TX 76402, USA; 40 Department of Microbiology, University of Lagos, University Road, Lagos, Lagos 101212, Nigeria; 41 Biology, Columbia State Community College, 1665 Hampshire Pike, Columbia, TN 38401, USA; 42 Department of Science, Neumann University, One Neumann Drive, Aston, PA 19014, US; 43 Molecular, Cellular, and Developmental Biology, Universtiy of Colorado, Boulder, 347 Colorado Ave, Boulder, CO 80309, USA; 44 Biology - School of Sciences, University of Louisiana at Monroe, 700 University Avenue, Monroe, LA 71209, USA; 45 Biology, Johns Hopkins University, 3400 N Charles St, Baltimore, MD 21218-2625, USA; 46 Biology Department, Oregon Coast Community College, 400 SE College Way, Newport, OR 97366, USA; 47 Science, Durham Tech, 1305 Antler Point Drive, Durham, NC 27713, USA; 48 Microbiology, Immunology, and Molecular Genetics, University of California, Los Angeles, 609 Charles E Young Dr E, Los Angeles, CA 90024, USA; 49 Biology Department, Winthrop University, 202 Dalton Hall, Rock Hill, SC 29733, USA; 50 Chemistry and Physics, Western Carolina University, 460 Memorial Drive, Cullowhee, NC 28723, USA; 51 Department of Biology, Colorado State University-Pueblo, 2200 Bonforte Blvd, Pueblo, CO 81001, USA; 52 Biology Department, South Texas College, 3201 Pecan Blvd, McAllen, TX 78501, McAllen, TX 78542, USA; 53 Biology, Tufts University, 200 College Ave, Robinson Hall, Medford, MA 02155, USA; 54 Biological and Chemical Sciences, New York Institute of Technology, 500 Northern Blvd, Old Westbury, NY 11568, USA; 55 Biological Sciences and Geology, Queensbrough Community College, 222-05, 56th Avenue, Bayside, NY 11364, USA; 56 Department of Biological Sciences, Idaho State University, 921 South 8th Avenue, Pocatello, ID 83209, USA; 57 School of Biology, University of St Andrews, Biomedical Sciences Research Complex, St Andrews, KY16 9ST, United Kingdom; 58 Biology, Gonzaga University, 502 E Boone AVE, Spokane, WA 99258, USA; 59 Science Division, Allegany College of Maryland, 12401 Willowbrook Rd, Cumberland, MD 21502, USA; 60 School of Molecular Biosciences, Washington State University, 370 Lighty Student Services Bldg, Pullman, WA 99164, USA; 61 Basic and Clinical Sciences, Albany College of Pharmacy and Health Sciences, 106 New Scotland Ave, Albany, NY 12208, USA; 62 Life Sciences, University of New Hampshire Manchester, 88 Commercial St, Manchester, NH 03101, USA; 63 Center for Biological Data Science, Virginia Commonwealth University, 1015 W. Floyd Ave, Richmond, VA 23284, USA; 64 Biology, Bryn Mawr College, 101 N. Merion Ave., Bryn Mawr, PA 19010, USA; 65 Biological Sciences, Lehigh University, 111 Research Dr., Bethlehem, PA 18015, USA; 66 Math and Natural Sciences, Collin College, 2800 E Spring Creek Pkwy, Plano, TX 75074, USA; 67 Biology Department, University of Wisconsin-River Falls, 410 South Third Street, River Falls, WI 54022, USA; 68 Biology, Saint Joseph’s University, 5600 City Ave, Philadelphia, PA 19131-1308, USA; 69 Department of Biological Sciences, University of Mary Washington, 1301 College Ave., Fredericksburg, VA 22401, USA; 70 Biology, Virginia Western Community College, 3080 Colonial Ave, Roanoke, VA 24015, USA; 71 Life Sciences Department/STEM Division, Harford Community College, 401 Thomas Run Road, Bel Air, MD 21015, USA; 72 Department of Biology, Austin Peay State University, 601 College Street, Clarksville, TN 37044, USA; 73 Science, Cabrini University, 610 King of Prussia Road, Radnor, PA 19087, USA; 74 Integrated Science, Business and Technology, La Salle University, 1900 West Olney Avenue, Philadelphia, PA 19141, USA; 75 Natural Sciences, Mount Saint Mary College, 330 Powell Avenue, Newburgh, NY 12550, USA; 76 Department of Biology, University of Evansville, 1800 Lincoln Avenue, Evansville, IN 47722, USA; 77 Department of Biological Sciences, Florida Gulf Coast University, 10501 FGCU Blvd. South, Fort Myers, FL 33965, USA; 78 Science and Mathematics, Northern State University, 1200 S. Jay St., Aberdeen, SD 57401, USA; 79 Molecular and Biomedical Sciences and Honors College, University of Maine, 5735 Hitchner Hall, Orono, ME 04469-5735, USA; 80 Hicks Honors College, University of North Florida, 1 UNF Drive, Building 9, Suite 2303, Jacksonville, FL 32224, USA; 81 Departamento de Microbiología Básica, Facultad de Ciencias Bioquímicas y Farmacéuticas-UNR, Suipacha 531, Rosario, Santa Fe 2000, USA; 82 Natural Sciences, College of Coastal Georgia, One College Drive, Brunswick, GA 31520, USA; 83 Biological Sciences, SUNY Old Westbury, 223 Store Hill Road, Old Westbury, NY 11568, USA; 84 Department of Biology, Thiel College, 75 College Avenue, Greenville, PA 16125, USA; 85 College of Health and Natural Sciences, Franklin Pierce U, 40 University Dr, Rindge, NH 03458, USA; 86 STEM Academy, Salish Kootenai College, 58138 US-93, Pablo, MT 59855, USA; 87 Department of Biology, Radford University, 501 Stockton St, Radford, VA 24142, USA; 88 Biological Sciences, Benedictine University, 5700 College Road, Lisle, IL 60532, USA; 89 Plant Pathology, Entomology, and Microbiology, Iowa State University, Science Hall, 2237 Osborn Dr., Ames, IA 50011, USA; 90 Biological sciences, Bowling Green State University, 129 Life Science, Bowling Green, OH 43403, USA; 91 Molecular Biosciences, University of South Floria, 4202 E Fowler Ave, SCA2015, Tampa, FL 33620, USA; 92 Biological Sciences, Webster University, 470 E Lockwood Ave, St. Louis, MO 63119, USA; 93 Department of Biology, Frostburg State University, 101 Braddock Rd, Frostburg, MD 21532, USA; 94 Institute for Advanced Medical Research and Training, University of Ibadan, College of Medicine, UCH Ibadan, Ibadan, Oyo 200113, USA; 95 Biology, Ouachita Baptist Univeristy, 410 Ouachtia Street, Biology Departmen, Biology Department, Arkadelphia, AR 71998, USA; 96 Department of Biology, Western Kentucky University, 1906 College Heights Ave., Bowling Green, KY 42101, USA; 97 Biology, University of Massachusetts, Amherst, 611 N. Pleasant Street, Amherst, MA 01003, USA; 98 Université Paris-Saclay, CEA, CNRS, Institute for Integrative Biology of the Cell (I2BC), Gif-sur-Yvette, 91190, France; 99 Ottawa Institute of Systems Biology and Deaprtment of Biochemistry, Michobiology and Immunulogy, University of Ottawa, 451 Smyth Rd., Ottawa, ON K1H8M5, USA; 100 Biology department, SUNY Plattsburgh, 101 Broad St, Plattsburgh, NY 12901, USA; 101 Natural Sciences, University of Houston-Downtown, 1 Main Street, Houston, TX 77002, USA; 102 Department of Biology, Washington University in St. Louis, One Brookings Dr, St. Louis, MO 63130, USA; 103 Science, Penn State Schuylkill, 200 University Ave, Schuylkill Haven, PA 17972, USA; 104 Biology, Salem State University, 352 Lafayette St., Salem, MA 01970, USA; 105 School of Life Sciences, University of Nevada, Las Vegas, 4505 Maryland Parkway, Las Vegas, NV 89154-4004, USA; 106 Biological Sciences, Southern Maine Community College, Fort Road, South Portland, ME 04106, USA; 107 School of Integrated Sciences, James Madison University, 800 S. Main, Harrisonburg, VA 22802, USA; 108 Biology Department, College of St. Scholastica, 1200 Kenwood Ave., Duluth, MN 55811, USA; 109 Biology, Carthage College, 2001 Alford Park Drive, Kenosha, WI 53140, USA; 110 Biology, Northwestern College, 101 7th St SW, Orange City, IA 51041, USA; 111 Biology Department, University of Puerto Rico at Cayey, 205 Antonio R. Barcelo Ave., Cayey, Puerto Rico 00736, Cayey, PR 00736, USA; 112 Biology, Case Western Reserve University, 2080 Adelbert Rd, Cleveland, OH 44106, USA; 113 Science and Math, Durham Technical Community College, 1637 E Lawson Street, Durham, NC 27703, USA; 114 Department of Biology and Chemistry, Alliance University, 2 Washington St, New York, NY 10004, USA; 115 Biology Department, The College of St. Scholastica, 1200 Kenwood Ave, Duluth, MN 55811, USA; 116 Biochemistry, Microbiology and Immunology, University of Otttawa, 850 crois. Peter Morand Cres., Ottawa, ON K1G 5Z3, USA; 117 Natural Sciences, CCSNH-Concord’s Community College, 31 College Dr., Concord, NH 03301, USA; 118 Translational and Molecular Medicine, University of Ottawa, 451 Smyth Rd., Ottawa, ON K1H8M5, USA; 119 Center for the Advancement of Science Leadership and Culture, Howard Hughes Medical Institute, 4000 Jones Bridge Road, Chevy Chase, MD 20815, USA; 120 Department of Biology, James Madison University, 951 Carrier Drive, Harrisonburg, VA 22807, USA

## Abstract

Course-based research pedagogy involves positioning students as contributors to authentic research projects as part of an engaging educational experience that promotes their learning and persistence in science. To develop a model for assessing and grading students engaged in this type of learning experience, the assessment aims and practices of a community of experienced course-based research instructors were collected and analyzed. This approach defines four aims of course-based research assessment – 1) Assessing Laboratory Work and Scientific Thinking; 2) Evaluating Mastery of Concepts, Quantitative Thinking and Skills; 3) Appraising Forms of Scientific Communication; and 4) Metacognition of Learning – along with a set of practices for each aim. These aims and practices of assessment were then integrated with previously developed models of course-based research instruction to reveal an assessment program in which instructors provide extensive feedback to support productive student engagement in research while grading those aspects of research that are necessary for the student to succeed. Assessment conducted in this way delicately balances the need to facilitate students’ ongoing research with the requirement of a final grade without undercutting the important aims of a CRE education.

## INTRODUCTION

Recent educational initiatives in STEM are facilitating wide-spread implementation of course-based research experiences (CRE) because they increase persistence for students across many demographics (Russell et al., 2007; Jordan et al., 2014; Hanauer et al., 2017; Hernandez et al., 2018). This educational approach is characterized by having students involved in conducting and contributing to authentic scientific research projects (Hanauer et al., 2006, 2012, 2016, 2017; Hanauer and Dolan, 2014; PCAST, 2012; Graham et al., 2013; Auchincloss et al., 2014; Hernandez et al., 2018). Recent research on the pedagogical approach to teaching a CRE describes how this educational design transitions the ways in which instructors teach and the way in which the relationship between the instructor and the student is conceptualized and manifest (Hanauer et al., 2022). In particular, the hierarchy which is so prevalent in most educational settings is flattened slightly with the instructor and student working together on a shared research project (Hanauer et al., 2022). The expertise of the instructor is utilized in supporting a research process, the outcomes of which are not necessarily known (Auchincloss et al., 2014). For both instructor and student, the research is on-going and to a degree unpredictable. Timing for various outcomes may vary across students and projects, the type of interaction and expertise that the instructor has to provide may change and broadly the instructor and student need to be flexible in the ways in which they interact around the emerging scientific work. Hanauer et al., (2022) describe in detail the nature of this pedagogy and the ways in which instructors work with students in teaching a CRE.

While the pedagogical implementation of a CRE transitions the relations between instructor and student, the institutional requirement for a grade has not changed. Classroom grading is a significant and ubiquitous practice in STEM education in general and is a requirement whether the class is a CRE or not. The specific nature of a CRE raises several problems in relation to classroom grading. How does a teacher maintain the process of “shared” scientific research that is important beyond the classroom, if the instructor is “grading” the student on in-class tasks? When the nature of a class is not dictated by delimited content knowledge or a prescribed set of skills, what are the aims of assessment within a CRE? How does an instructor support and encourage a student during the challenges and potential failures of authentic science, if both student and instructor know that they need to assign a grade for the work being conducted? Broadly the problem of assessing and grading students in a CRE is that the CRE aims to provide a professional, authentic research experience in which the student feels that they are scientists. Grading seems quite artificial in this particular educational design.

Prior approaches to assessing a student’s scientific inquiry divide into two camps: analytic schemes and authentic task modelling. Early work used an analytic scheme to define the components of scientific inquiry and suggested methods for assessing each of the parts in isolation. For example, Zachos (2004) delineates the core capabilities of scientific inquiry to include coordinating theories, searching for underlying principles, being concerned with precision, identifying sources of error in measurement and proportional reasoning, and suggest these should be used in the design of a series of performance tasks. Wenning (2007) designed a multiple-choice test of the components of a scientific inquiry such as identifying a problem, formulating a hypothesis, generating a prediction, designing an experiment, collecting and organizing data, using statistical methods and explaining results. Shavelson et al., (1998) proposed using a range of performance tasks to evaluate scientific inquiry abilities of students. In line with this analytic approach, Palaez et al., 2017 specified a set of core experimentation competencies consisting of the categories - identify, question, plan, conduct, analyze, conclude and communicate. Zelaya, Blumer & Beck (2022) categorize 14 survey style instruments and 16 evaluation rubrics in relation to this set of competencies specifying the degree of overlap between each tool and the specified competencies. Similarly, in an extensive review of the existing tools that can be used for the assessment of a CURE, Shortlidge & Brownell (2016) review 26 survey style tools that can be used to assess different aspects of the research experience such as critical thinking, views of science, project ownership, biological concepts and experimental design. What many these approaches have in common is the idea that the grading of scientific inquiry can be externalized from the actual research that the student is doing; students are evaluated for a set of skills, competencies, dispositions and abilities for future scientific research.

The second camp proposed modelling authentic activity. In principle, if a CRE involves authentic research which produces scientific findings useful for a scientific community and the student is seen as a researcher, it would be logical that the evaluation of the student’s work would be situated in the ways professional scientists are evaluated. However, practically, waiting for a paper to be published or a poster presented at a professional conference would be problematic both in relation to timing and the threshold level for successful student outcomes. Instead, Hanauer, Hatfull & Jacobs-Sera (2009) proposed an approach termed *Active Assessment* which analyzes the professional research practices of a specific research project and then uses these as a way of generating a rubric for evaluating student work. Assessment is done on the student as they work through the scientific inquiry they are involved in. A similar approach has been proposed by Dolan and Weaver (2021). What characterizes this approach are the ideas that assessment and grading should be situated in the performance of a student while conducting research in the CRE and that this assessment should be based on professional performance.

However, while this second approach offers a conceptual basis of how assessment in a CRE could be conducted, it is not based on data from actual instructors teaching a CRE. The aim of this study is to look at how experienced instructors in a large-scale CRE program -- the Science Education Alliance (SEA) program by the Howard Hughes Medical Institute (HHMI) – describe their processes of assessing their students engaged in course-based research. Working with this large community of experienced CRE instructors over a two-year period, models of CRE assessment were developed. In addition, this current paper builds upon prior research on models of CRE instruction, which were similarly developed with this community of SEA instructors, (Hanauer, et al., 2022). The outcome of this study thus provides insight into how CREs can be assessed and graded while maintaining the pedagogical approach designed to provide an authentic research experience for students and enhance persistence.

### Issues with Assessment and Grading

In a classic text, Walvoord and Anderson (1998) specify a series of basic roles that grading is expected to perform: 1) It should be a reliable measure of a student’s performance of required work; 2) It should be a means of communicating the quality of the student’s performance with parents, other faculty, the university, future institutions and places of work; 3) It should be a source of motivation; 4) It should provide meaningful information for feedback to students and instructors to enhance learning; and 5) It can be a way of organizing class work. However, as seen in the scholarship, the implementation of grading is not unproblematic.

As documented over decades, there are questions as to whether grading always fulfills the stated aims above (Jaschik, 2009). Prior research has suggested that STEM faculty have the knowledge to create assessment tasks but often lack an understanding of how to validate these tasks (Hanauer & Bauerle, 2015). Some faculty problematically assume that the way they were graded is a basis for the grading of their own students leading to a persistence of outdated assessment practices (Boothroyd & McMorris, 1992). When considering what to assess and grade, there can be confusion between learning components tied to stated learning objectives of the course and other aspects of being a student such as punctuality, attendance, and participation (Hu, 2005). Additionally, there is little agreement between instructors as to which components should go into a grade with different instructors varying greatly in relation to how assessment is conducted (Cizek, Fitzgerald & Rachor, 1996). Research has also shown that grades can vary in relation to variables such as instructors, departments, disciplines and institutions (Lipnevich, et al., 2020) and in relation to specific student characteristics such as physical attractiveness (Baron & Byrne, 2004) and ethnicity (Fajardo, 1985).

It is important to understand the central role grading plays in the lives of students. Grading can increase anxiety, fear, lack of interest and hinder the ability to perform on subsequent tasks (Butler, 1988; Crooks, 1988, Pulfrey et al., 2011). There are alarming rates of attrition from STEM documented for students who identify as African American or Black, Latino or Hispanic, and American Indian and Alaska Native (Asai, 2020; Whitcomb & Chandralekha, 2021; National Science Board, 2018) and low grades is one of the factors that leads to this outcome (Whitcomb & Chandralekha, 2021). The relationship between grading and persistence is situated in the effect of negative feedback on performance (such as a lower-than-expected grade) and the individual’s sense of self-efficacy in that field (Bandura, 1991, 2005). Students who identify as African American or Black, Latino or Hispanic, and American Indian and Alaska Native may enter the STEM fields with pre-existing fears and anxieties about their work resulting from stereotype threat (Hilts et al., 2018). Negative experiences with grading further exacerbate these feelings leading to a disbelief in their ability to continue in STEM and hence attrition from that course of study (Hilts et al., 2018; Whitcomb & Chandralekha, 2021). Recent research has shown that grading works in two parallel ways: lower grades limit the opportunities that are available to students and increase the negative psychological impact on students’ intent to persist in STEM (Hatfield, Brown & Topaz, 2022). As such grading, if not conducted appropriately, could directly undermine the main aim of a CRE – increased persistence in STEM for all students.

## METHODOLOGY

### Overview:

A multi-method, large-scale and multi-year research methodology was employed in this study. Data collection and analysis was conducted over a two-year period in a series of designed stages with full participation from a large group of CRE instructors and a dedicated science education research team. The project developed in the following stages:

1. *Survey*: The initial stage of the study involved a qualitative and quantitative survey. The qualitative section asked about grading and assessment procedures used by instructors in their CRE courses and asked for a detailed explanation of the way these were used in their courses. The quantitative section used the psychometrically validated scales of the Faculty Self-Reported Assessment survey (Hanauer and Bauerle, 2015) to evaluate the knowledge level of the surveyed faculty. The aim of this first stage of the project was to collect descriptive data on the participants’ understanding of assessment and specific information on the way they conduct assessment and grading in their courses.
2. *Analysis and Large-Scale Community Checking of Assessment Aims and Practices*: Data from the qualitative study was analyzed using a systematic content analysis process and the quantitative data was analyzed using standard statistical procedures. The quantitative data was analyzed in terms of high-level assessment aims and specific grading and assessment practices. All analyses were summarized and then presented in a workshop setting to a cohort of 106 CRE instructors. In a small-focus group format, the aims and practices were presented and instructors provided written feedback on the validity of the analysis, the specification of the high-level aims, the specification of practices and the assignment of the practices to assessment. Instructors responded within the workshop and were subsequently given an additional week to provide online responses to the questions posed. All data was collected using an online survey tool.
3. *Analysis and Community Checking of Models of Assessment and Grading*: Data from the first stage of community checking was analyzed for modifications to the assessment aims and the assigned assessment and grading practices. Percentage of agreement with the aims and practices was calculated and modifications to the models were assigned. During this analysis there were no changes to the high-level aims, but several specific practices were added. Once the table of aims and practices had been finalized, the original survey commentary dealing with how assessment and grading were conducted was consulted. Using this commentary and the pedagogical models of CRE instruction (Hanauer et al., 2021), the aims and practices of assessment were integrated with the discussion of CRE instruction. Three integrated models were developed and presented to a dedicated group of 23 instructors for validation process. Instructors were asked to provide feedback on the quality and descriptive validity of the models, the specification of aims of assessment and the specific practices. Instructors provided feedback during the workshop and for a week after the workshop. All data were collected using an online survey tool.
4. *Finalization of the Models*: Feedback from the workshop was analyzed for verification of the models and any required modifications that might be needed. Agreement with the models and their components were checked. Following this process, the models were finalized.

### Participants:

Participants for this study were elicited from the full set of instructors who teach in the SEA program. The SEA program is a large-scale, two semesters, program implemented at 190 institutions predominantly with Freshman and Sophomore students. This course is supported by the Howard Hughes Medical Institute and has scientific support from the Hatfull laboratory at the University of Pittsburgh. For the first stage of data collection, a survey request was sent to 330 SEA instructors. 105 faculty responded with 72 instructors providing full answers on the survey. Table 1 presents the instructor demographics. The SEA faculty respondents are predominantly White (≥58.1%) and women (≥49.5%). A range of academic ranks from instructor to full professor were represented in the sample. As seen in Table 1, the majority of respondents had at least three years of teaching in the program and above 6+ years of teaching postsecondary science. Respondents for the community checking of the model were drawn from the SEA faculty. For each stage 100+ instructors participated. Demographic data was not collected on the participants at the 2 community checking sessions. As a community of CRE instructors, during the semester, the SEA has a weekly 1-hour, Friday afternoon session providing scientific and educational instructor development. During the Fall 2022 semester, two sessions were conducted by the Lead Assessment Coordinator of the SEA (Dr. Hanauer) dedicated to the development of a meaningful assessment approach. The sessions involved a lecture approach of general principles of assessment including constructive alignment between objectives and instruments, active assessment instruments that could be used and ways of interpreting outcomes. Participation in these Friday sessions were voluntary. Approximately 50 faculty attended these two sessions.

### Instruments:

As described in the overview of the research process, data collection consisted of a qualitative and quantitative initial survey, followed by a large community checking survey and a final assessment model checking survey. A specific tool was developed for each of these stages. The original survey consisted of three sections:

1. ***Familiarity with Assessment Terms***: The first set of items were from the psychometrically validated Faculty Self-Reported Assessment survey (Hanauer & Bauerle, 2015). The survey consists of 24 established terms relating to assessment, organized into two components – assessment program and instrument knowledge, and knowledge of assessment validation procedures. On a 5-point scale of familiarity (1=I have never heard this term before; 5=I am completely familiar with this term and know what it means), faculty rated each of the terms in relation to their familiarity with the term. The FRAS is used to evaluate levels of experience and exposure of faculty to assessment instruments and procedures. See Table 2 for a full list of the assessment terms used.
2. ***Qualitative Reporting of Student Assessment:*** The second set of items were qualitative and required the instructor to describe the way in which they assess students in the SEA program, to specify the types of assessment used (such as quiz, rubric…etc.), and to explain what each assessment is used for. Following the first question, faculty were asked to describe how they grade students and what goes into the final grade. Answers consisted of written responses.
3. ***Self-Efficacy Assessment Scales***: The third set of items consisted self-reported measures of confidence in completing different aspects of assessment. The 12 items were taken from the FRAS (Hanauer & Bauerle, 2015) and consisted of a set of statements about the ability to perform different aspects of the assessment process (see Table 3 for a full list of the statement). All statements were rated on an agreement scale (1=Strongly Disagree, 5=Strongly Agree).

In order to collect verbal responses during the community checking stage of this project, participants completed an online survey that was presented following a shared online session in which the analyses of the main aims of assessment and the associated practices were presented (see Table 3). The survey asked for a written response to the following questions relating to each of the specified aims and associated practices:

1. Does this assessment aim make sense to you? Please specify if you agree or disagree that this is an aim of your CRE assessment.
2. For this aim, do the practices listed above make sense to you? Please comment on any that do not.
3. For this aim, are there practices of assessment that are not listed? If so, please list these additional practices and describe what these practices are used to evaluate.
4. Are there aims of assessment beyond the 4 that are listed above? If so, please describe any additional aims of assessment below.

**Figure 1:**
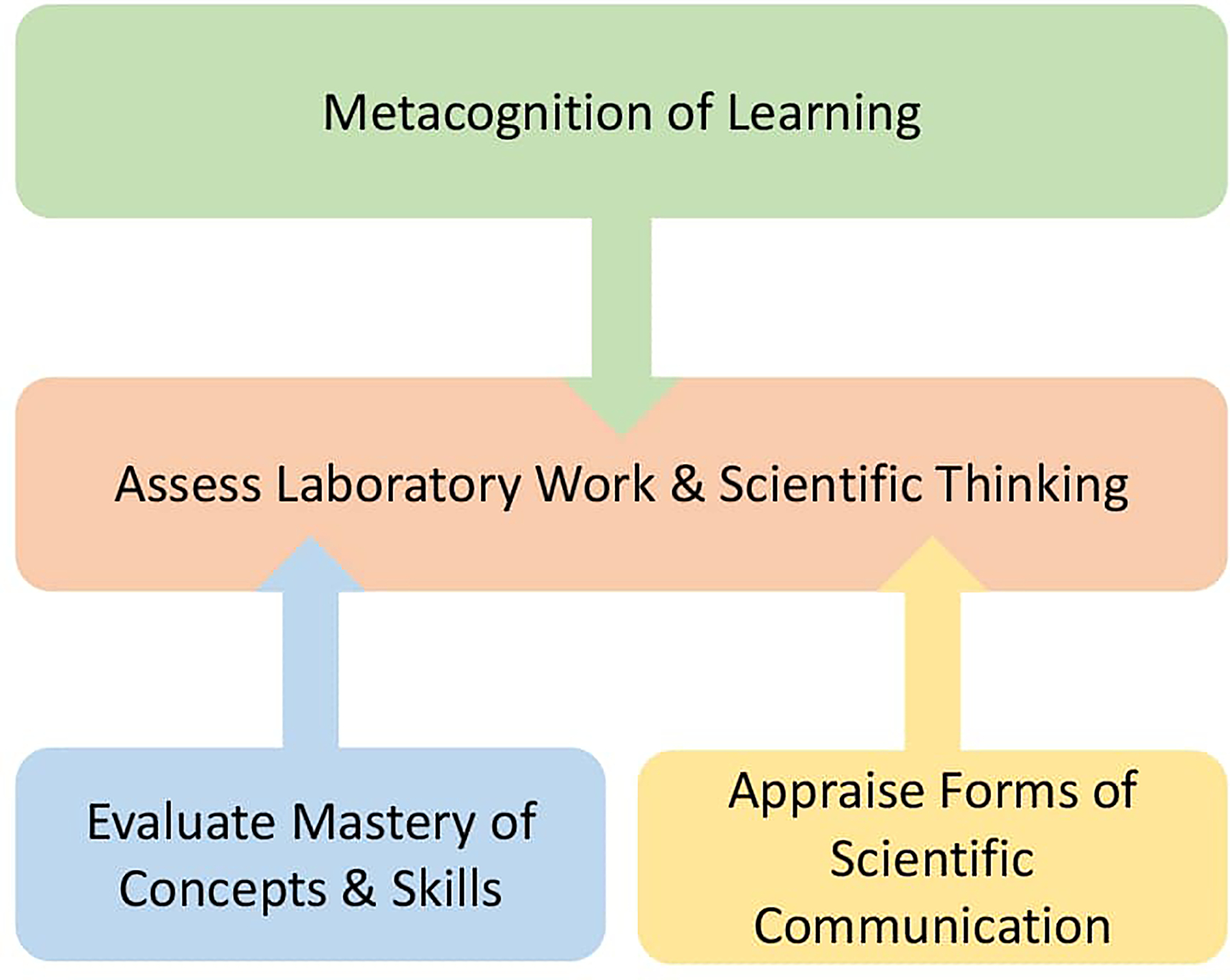
The Core Components of a CRE Assessment Model: Based on the qualitative analysis of faculty descriptions of their assessment and grading practices in a CRE, four central aims of assessment were defined: 1. Assess Laboratory Work and Scientific Thinking; 2. Evaluate Mastery of Concepts, Quantitative Thinking, and Skills; 3. Appraise Forms of Scientific Communication; & 4. Metacognition of Learning. Together these four aims and associated assessment and grading practices define the assessment program of a CRE.

**Figure 2:**
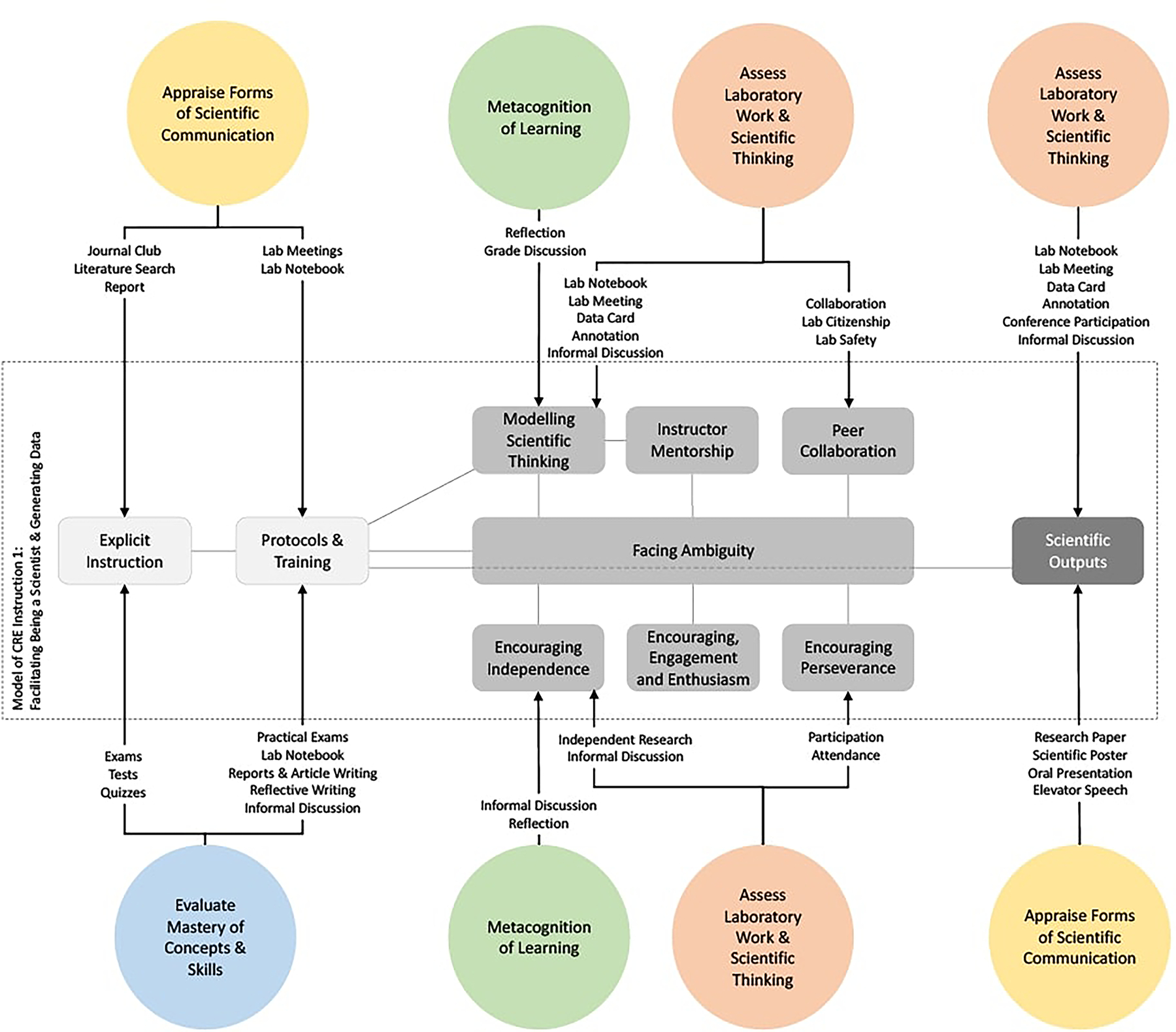
Assessing Being a Scientist and Generating Data: This model has three distinct stages. The first stage relates to the assessment of implicit instruction and protocol training. The second stage relates to aspects of doing science in the laboratory and the final stage relates to scientific outputs. The model presents the aims and practices of assessment applied at each of these stages.

**Figure 3:**
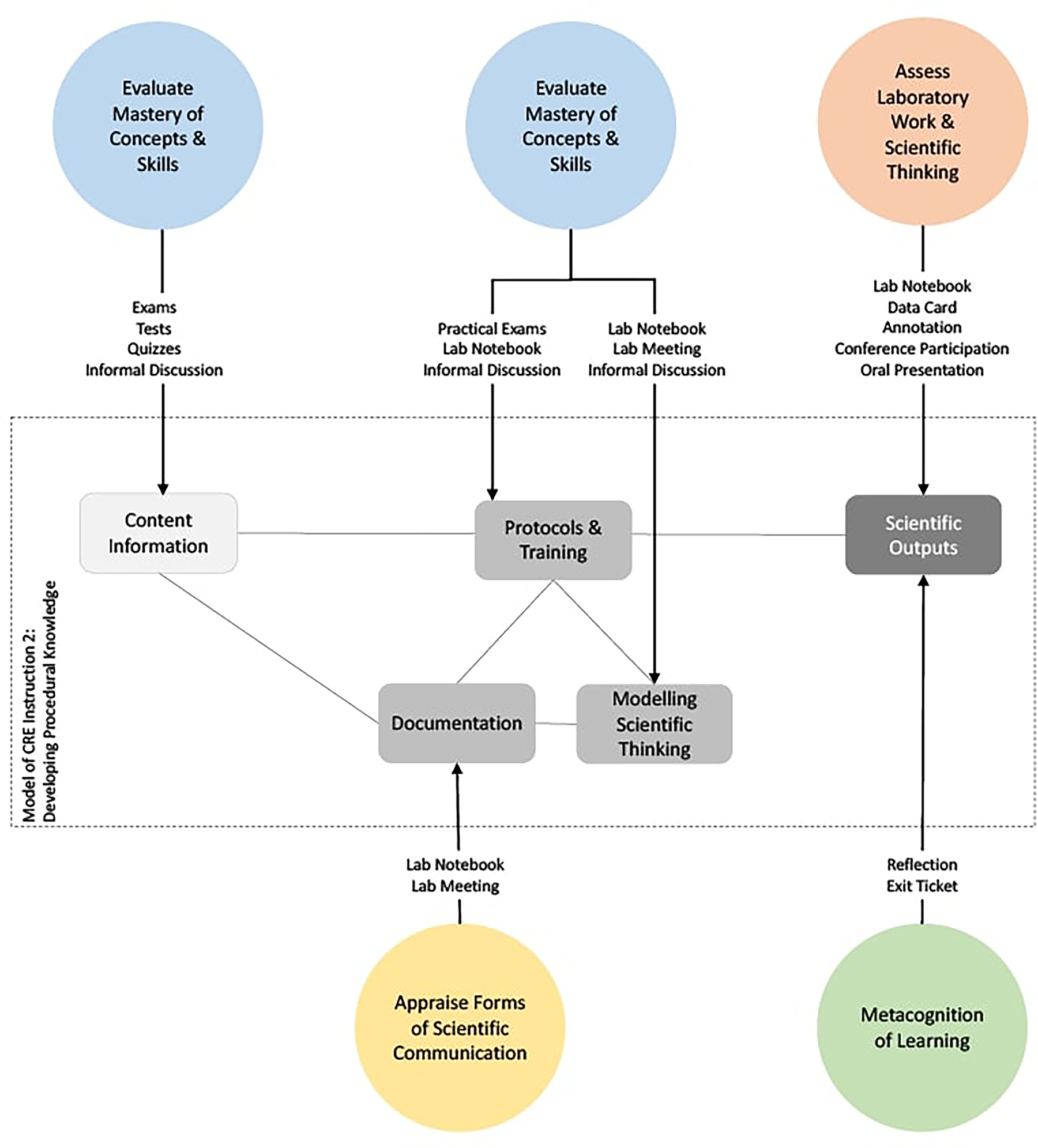
Assessing Procedural Knowledge: This model has three distinct stages. The first stage relates to content information. The second stage relates to protocol training and training a student to think like a scientist. The third stage relates to scientific outputs. The model presents the aims and practices of assessment applied at each of these stages.

The final community checking procedure involved the presentation of the full models of assessment to the collected participants in a shared online session (see Figures 1, 2 and 3). Following the presentation of the models, the participants were divided into groups and each group was assigned a model to discuss and respond to. Each model was reviewed by two groups, and all responses were collected using an online written survey with the following questions:

1. For each of the instructional models, have the appropriate assessment aims been specified?
2. For each of the instruction models, have the appropriate assessment practices been specified?
3. Overall, do the models present an accurate and useful description of grading practices in the SEA?
4. Please suggest any modifications and comments you have on the model.

### Procedures:

Data was collected in three stages. The initial stage consisted of an online survey that was distributed to all faculty of the SEA using the web-based platform Qualtrics. Following the informed consent process responses to the qualitative and quantitative items were recorded. The second stage involved the collection of community checking data from SEA instructors. A dedicated online Zoom session was arranged for this during one of the monthly virtual faculty meetings organized through the SEA program. During a one-hour session the analysis of the aims of assessment and the associated practices were presented to the faculty. In small groups (breakout rooms), each of the aims and its associated practices were discussed. Following the session, an online survey was sent to faculty to collect their level of agreement with the aims and practices that were presented. They were also asked to modify or add any aims or practices that had been missed in the presented analysis of the original survey. The third stage of community checking data analysis consisted of a second online session during the regular end- of- week faculty meeting. During a one-hour session, each of the assessment models was presented to the faculty who then discussed them in small groups (breakout rooms). A survey was sent to the faculty during the session to respond to the models and write their responses to the models. All data was collected in accordance with the guidelines of Indiana University of Pennsylvania IRB #21–214.

### Analysis:

The analysis of the data in this study was conducted in four related stages. The initial survey had both quantitative and qualitative data. The quantitative data was analyzed using established statistical descriptive methods. The qualitative verbal data consisted of a series of written statements relating to the practices used for assessment by the different instructors and the aims of using these practices. Using an emergent content analysis approach, each of the instructor statements was analyzed and coded. Two different initial code books were developed. One dealt with the list of practices used by the faculty; the second involved the explanation of why these practices were used and what the instructor was trying to assess. The data was coded by two trained applied linguistic researchers and following several iterations, a high level of agreement was reached on the practices and aims specified by the instructors. The second stage of this analysis of the verbal survey data consisted of combining the aims and practices codes. The specified practices across all of the instructors for each of the aims was tabulated. A frequency count of the number of faculty who specified each of the practices was conducted. The outcome of the first stage of analysis was a statistical description of the levels of knowledge and confidence of faculty on assessment issues and the specification of four main aims of assessment with associated assessment practices.

The second stage of analysis followed the presentation of the tabulated coded data from the original survey to participants. In this stage of community checking, faculty specified agreement (or disagreement) with the assessment aims and the set of associated practices. The verbal responses were analyzed by two applied linguistics researchers and modifications were made to the tabulated data. The degree of agreement with each of the aims and associated practices was counted. Any additional practices specified by faculty were added to the model. No new aims were specified and as such no changes were made. The table of assessment aims and practices was finalized.

Having established the aims of assessment and related practices, a third stage of analysis involved integrating the emergent assessment aims and practices with models of CRE instruction which had been previously defined for the SEA instructors (see Hanauer et al., 2022 for full details). A team of two researchers worked together to specify the points of interaction between the instructional and assessment components of CRE teaching. Using the qualitative data of the original models and the verbal statements of aims for the assessment data, integrated models of assessment were developed. Following several iterations, three assessment models corresponding to the instructional models were specified.

The final stage of analysis followed the presentation of the models of assessment to the community of SEA faculty. A team of two researchers went over the changes presented by faculty in relation to each of the models. Changes that were specified, such as the addition of specific practices into different models, were made. The outcome of this process was a series of three models that capture the aims and practices of assessment.

## RESULTS

### Instructor Familiarity and Self-Efficacy with Assessment

To build models of CRE assessment based on qualitative reports from instructors in the SEA program, we first evaluated instructors’ knowledge of assessment terms and their confidence in implementing assessment tasks. For instructor knowledge of assessment, we utilized the Faculty Self-Reported Assessment Survey (FRAS) (Hanauer and Bauerle, 2015) – a tool which measures two components of assessment knowledge: 1) knowledge of assessment programs and instruments and 2) knowledge of assessment validation. Internal consistency was calculated for the each of the FRAS components. Cronbach’s Alpha was 0.86 for the Knowledge of assessment programs and instruments components and 0.94 for the knowledge of assessment validation component. These levels suggest that each of the components is sufficiently consistent and hence reliable.

For the Program and Instrument component, instructors reported high levels of familiarity (Scale = 1 – 5, Grand Mean= 4.26, Std. = 0.55). All items were above 4 (high level of familiarity), except for the terms related to performance assessment. These latter terms, which include Alternative Assessment and Authentic Assessment, were nevertheless familiar to instructors (above 3). The Validation components of the survey, which addresses terms relating to the evaluation and quality control of assessment development, were also familiar to instructors (Grand Mean = 3.34, Std. = 0.35). This result is in line with prior studies of faculty knowledge of assessment terms (Hanauer and Bauerle, 2015). The results overall for the two dimensions suggest that instructors in this study have the required degree of assessment understanding to be reliable reporters of their assessment procedures and activities.

To augment the FRAS data, self-efficacy data was collected on instructors’ confidence in completing assessment related tasks. Internal consistency was calculated for the self-efficacy scale. Cronbach’s Alpha was 0.93 which shows that this scale is reliable As shown in Table 3, instructors reported high levels of confidence in their assessment abilities (Scale = 1 – 5, Grand Mean =4.04, Std. =0.65). The highest confidence was in relation to defining important components of their course and student learning outcomes, while the lowest levels of confidence were in relation to the ability to evaluate, analyze and report on their assessments. The confidence levels for the latter were still relatively high (just below 4) and reflect, to a certain extent, the same trend as seen using the FRAS instrument. Taking into consideration the results of the FRAS and self-efficacy tasks, instructors report moderate to high levels of assessment expertise and confidence, which suggest that these instructors have the required expertise to report and evaluate the aims, practices and models of CRE assessment.

### Aims and Practices of CRE Assessment

A fundamental goal of this study was to describe the aims and practices of experienced CRE instructors for assessing students in a CRE. As described in the methodology section, a list of aims and practices for assessment was elicited from the written survey data completed by instructors in the HHMI SEA program, which was then community-checked and modified. The faculty were asked to describe how they assess students in the SEA program what types of assessment used (such as quiz, rubric…etc.), and to explain what each assessment is used for. The aims specified by the faculty reflected components of pedagogical activity that came together while teaching a CRE. So, for example, assessing the physical work of lab was integrated with scientific thinking as a single aim. Broadly the aims reflected work in the laboratory, aspects of mastery, communication and student self-evaluation of their learning

4 central aims of CRE assessment were defined. For each aim, there were a cluster of assessment practices that were employed to assess student learning, with different instructors utilizing different subsets of these practices. The aims of CRE assessment, the practices related to each of the aims, and the degree of agreement amongst faculty for each aim and set of practices are presented in Table 4 and described below:

1. ***Assess Laboratory Work and Scientific Thinking:*** The objective of this assessment aim was to assess a student’s readiness, in terms of their practices, thought patterns and ethics, to function as a researcher in the laboratory setting. As seen in Table 4, several different practices were related to this aim, which include 1) assessing student behaviors such as participation, attendance, citizenship, collaboration, safety and independence, and 2) assessing students’ scientific thinking based on their lab notebooks, data cards, independent research, conference participation and informal discussion. During the community checking stage, 85.95% of the faculty specified that this category was an aim of their assessment program and that the assigned practices were appropriate.
2. ***Evaluate Mastery of Concepts, Quantitative Thinking, and Skills***: The objective of this assessment aim was to assess the underpinning knowledge and skills that students need in order to function successfully, as a researcher, in the CRE laboratory setting. The practices related to this assessment aim include 1) the checking of laboratory techniques and skills using practical exams and lab notebooks, 2) the evaluation of required scientific knowledge through exams, tests, quizzes, written reports and articles, and 3) the assessment of quantitative knowledge. During the community checking stage, 80.99% of faculty specified that this category was an aim of their assessment program and that the assigned practices were appropriate.
3. ***Appraise Forms of Scientific Communication***: The objective of this assessment aim was to evaluate the ability of students to convey their research and attain scientific knowledge through the different forms of science communication. The practices related to this assessment include 1) oral abilities such as oral presentation, peer review, lab notebook meetings, scientific poster and elevator speech, and 2) literacy abilities such as reading and writing a research paper, report writing, notebook writing, scientific paper reading, literature review, and poster creation. 63.64% of faculty specified that this category was part of their assessment program.
4. ***Metacognition of Learning***: The objective of this assessment aim was to assess the ability of students to regulate and oversee their own learning process. This aim is based on the assumption that being in control of your learning process improves the ability to learn. The practices related to this aim include reflection, discussion and an exit ticket. 76.85% of faculty specified that this category was part of their assessment program.

These four aims and associated practices define a program of assessment for CRE teaching. As depicted in Figure 1, the central aspect of an assessment program for a CRE is to evaluate the ability of a student to work and think in a scientific way. This central aspect is supported by two underpinning forms of knowledge: 1) mastery of concepts, quantitative thinking and skills and 2) the ability to communicate science. Overseeing the whole process is metacognition, which allows the student to regulate and direct their learning process. Accordingly, information on the students’ functioning across all these areas are collected as part of the assessment program.

### Models of Assessment in a CRE

The assessment program presented in this study is implemented by instructors in conjunction with a program of CRE instruction that has been previously described (Hanauer et al., 2022). The assessment aims and practices described here can therefore be integrated with the aims and practices (or models) of CRE instruction. The stated aims of CRE instruction are 1) Facilitating the experience of being a scientist and generating data; 2) Developing procedural knowledge, that is the skills and knowledge required to function as a researcher; and 3) Fostering project ownership, which include the feelings of personal ownership and responsibility over their scientific research and education (Hanauer, et al., 2022). These aims are directly in line with the broad aim of a CRE in providing a student with an authentic research experience (Dolan & Weaver 2021). In the sections that follow, and using a constructive alignment approach (Ambrose, et al, 2010; Biggs, 1996), the assessment aims and practices uncovered in this study are presented with the associated models of CRE instruction previously described.

#### Model 1: Assessing Being a Scientist and Generating Data

Being a scientist and generating novel data is a core aspect of a CRE. As shown in Figure 2 and described below, the instructional approach to achieving this aim involves three stages of instruction:

- Stage 1 involves preparing the student with the required knowledge and procedures in order to function as a researcher who can produce usable data for the scientific community. The pedagogy employed here includes the use of explicit instruction to provide students with the foundational knowledge to understand the science they are involved with and protocol training to make sure a student can perform the required scientific task.
  Accordingly, assessment in this first stage of the model is aimed at Evaluating Mastery of Concepts and Quantitative Thinking. The assessment practices used here include both exams and in class quizzes, which are well suited for this purpose. Additionally, given that this foundational scientific knowledge must often be retrieved from various forms of scientific communication, including lecture, a research paper, a poster and an informal discussion with an expert, the ability to use scientific communication for knowledge acquisition is also evaluated. Practices such as the evaluation of a literature search report or presentation at a journal club can provide information on how the student understands and uses different modes of scientific communication. Combined, the use of exams, quizzes, literature search reports and journal club participation can provide a rich picture of the foundational knowledge of a student as they enter the process of doing authentic research.
  To assess a student’s ability to use a range of specific protocol properly, instructors rely on practical exams and a student’s lab notebook, which are well established ways of checking whether a student understands and knows how to perform a specific procedure. Beyond these approaches, instructors reported that they used informal discussion, reflective writing, article writing and the lab notebook meeting to evaluate formally and informally whether the students understand how to perform the different scientific tasks that are required of them. This combination of explicit teaching of scientific knowledge and procedures, with formal and informal assessment of these abilities, serves to create a basis for the second stage of this pedagogical model, described below.
- Stage 2 involves supporting students to manage the process of implementing procedures in order to generate authentic data. A central aspect of this stage is that the student moves from a consumer to a producer of knowledge, and this involves a change in the students’ mindset concerning thinking processes, independence, perseverance and the ability to collaborate with others. Importantly, as is the case with science, positive results are not guaranteed and students face the ambiguity of failed outcomes and unclear paths forward. It is for this reason that the pedagogy at this stage involves a range of different supportive measures on the part of the instructor. These include modeling scientific thinking, providing encouragement and enthusiasm, mentoring the student at different points and, most importantly, making sure that the students understand that the scientific process is one that is fraught with challenges that need to be overcome. A lot of instruction is provided at the time that a task or event occurs.
  Assessment at this stage is covered by the aim of Assessing Laboratory Work and Scientific Thinking and the Metacognition of Learning. The scientific thinking of the student is primarily assessed through the discussion of the lab notebook, data and annotation cards, often during lab meetings. Importantly, as reported by faculty, a lot of this assessment is directed by informal discussion with the aim of providing direct feedback to the student so that they can perform the tasks that are required. This is very much a formative assessment approach with direct discussion with the student while they are working and in relation to the research they are doing. There are behaviors that faculty specify are important to track, such as participation, attendance, collaboration, lab citizenship and lab safety. These behaviors are a prerequisite for the research to move forward for the student and the research group as a whole. The use of assessment practices such as reflection and discussion allows the assessment of the degree of independence of the student, in addition to actually positioning the student as independent; the requirement of a reflection task, whether written in one’s lab notebook or verbally, situates the students as the researcher thinking through what they are doing. Overall, this stage involves extensive informal formative assessment of where the student is in the process from the practical, scientific and emotional aspects of doing science, combined with a more formal evaluation of the behaviors which underpin a productive and safe research environment.
- The third and final stage of this pedagogical model involves the actual scientific output produced by the student researcher. A CRE is defined by the requirement that data is produced that is actually useful for a broader community of scientists. If the second stage of the assessment of this pedagogical model is characterized by informal, formative assessment approaches, this final stage is characterized primarily by formal summative assessment. At this stage the student has produced scientific knowledge and is in the process of reporting this knowledge using established modes of scientific communication. The student is assessed in relation to the knowledge they have produced and the way they communicate it. As such, both the aims of Assessing Laboratory Work and Scientific Thinking and the Appraisal of Forms of Scientific Communication are utilized. The lab notebook, data card, annotation, conference presentation, oral presentation and poster all involve a double summative assessment approach: an evaluation of the quality of the scientific work that has been produced and an evaluation of the ability of the student to communicate this knowledge using established written and verbal modes of scientific communication. This final stage provides the opportunity for evaluating the whole of the research experience that the student has been involved in.

To summarize, the instruction and assessment model of Being a Scientist and Generating Data has three distinct stages. The initial stage is designed to make sure that the student can perform the required tasks and understand the underlying science. Assessment at this stage is important as the learning involved in this stage is a prerequisite for the second stage of the model. During the second stage, while the student is functioning as a researcher, the primary focus of the assessment model is to provide feedback to the student and the required level of expertise advice and emotional support to allow the research to move forward. This stage is characterized by informal discussion and is primarily a formative assessment approach. The final stage is directed at evaluating the scientific outcomes and the student’s ability to communicate them. Assessment at this stage offers a direct understanding of the quality of the work that has been conducted, the degree to which the student understands the work, and the ability of the student to communicate it.

#### Model 2: Assessing Procedural Knowledge

Being able to perform a range of scientific procedures is a central and underpinning aspect of being a scientist and a core feature of a CRE. Figure 3 presents a pedagogical and assessment model for teaching procedural knowledge. As seen in the previous model, protocols are an important precursor that enables an undergraduate student to conduct scientific research. In model 2, how students learn scientific procedures is further explicated from model 1. As can be seen in Figure 3, there are three stages to the development of procedural knowledge.

- The first stage involves enhancing the students’ content knowledge concerning the science behind the protocol they are using and scientific context of the research they will be involved with. For a student to become an independent researcher, they need to be able to not just follow a set of procedures but also to understand the science that it relates to. The pedagogical practice involved here includes explicit instruction, discussion and reading of primary literature. From an assessment perspective, the evaluation of this underpinning content knowledge is conducted using established practices such as exams, tests and quizzes. In addition, as reported by faculty, this material was informally discussed with students to gauge understanding of the context and role of the procedure.
- In the second stage, students are taught how to implement the procedure and to think like a scientist. This involves using a protocol, scientifically thinking through the process of using a protocol, and appropriate documentation of the process of using a protocol. Scientific thinking at this stage includes interpretation of outcomes, problem solving, and deciding about next steps. In this way, learning a protocol is not only about being able to perform, analyze and document a procedure appropriately, but also involves the development of independence for the researcher. These two components are related in that if a student really has a full understanding of the procedure, they can also make decisions and function more autonomously. Such mastery is particularly critical in a CRE because the research being conducted is intended to support an ongoing authentic research program. As reported by faculty, there are both formal and informal assessments that facilitate this evaluation. Practical exams allow faculty to really check the performance of a particular procedure and their understanding. Lab notebook evaluation, lab meeting interactions and informal discussion about the work of a student as they perform certain tasks provides further evidence of the student’s mastery of the concepts and skills that are involved. These interactions are primarily formative and have the aim of providing feedback for the improvement of the student’s understanding of scientific procedures.
  An additional level of assessment at this stage relates to the ability of students to document their research in the lab notebook, explain their research in a lab meeting and to converse with peers and instructors about what they are doing. These are all aspect of scientific communication, and assessment at this second stage of learning procedural knowledge includes the aims evaluating mastery of concepts and skills and of an appraisal of scientific communication. Since these are new forms of communication for many undergraduate students, instructors report using rubrics to evaluate and provide feedback on the quality of the communication.
- The final stage of this model relates to the scientific outcomes of the students’ work. At this stage, assessment aims to evaluate the quality of the outcomes of these procedures and the level to which the student really understands what they have done. Evaluation here therefore combines the use of data cards, annotation outputs, lab notebooks, oral presentations, conference participation, and the student’s reflections on their own work. As reported by faculty, not all procedures are successful and students are not graded negatively for a failed experiment as long as the procedures, including the thinking involved, follows the scientific process. Thus, as reported by faculty, both the instructor and the student often work collaboratively to evaluate how well the student understands the different procedures they are learning to use.

#### Model 3: Assessing the Facilitation of Project Ownership

**Figure 4:**
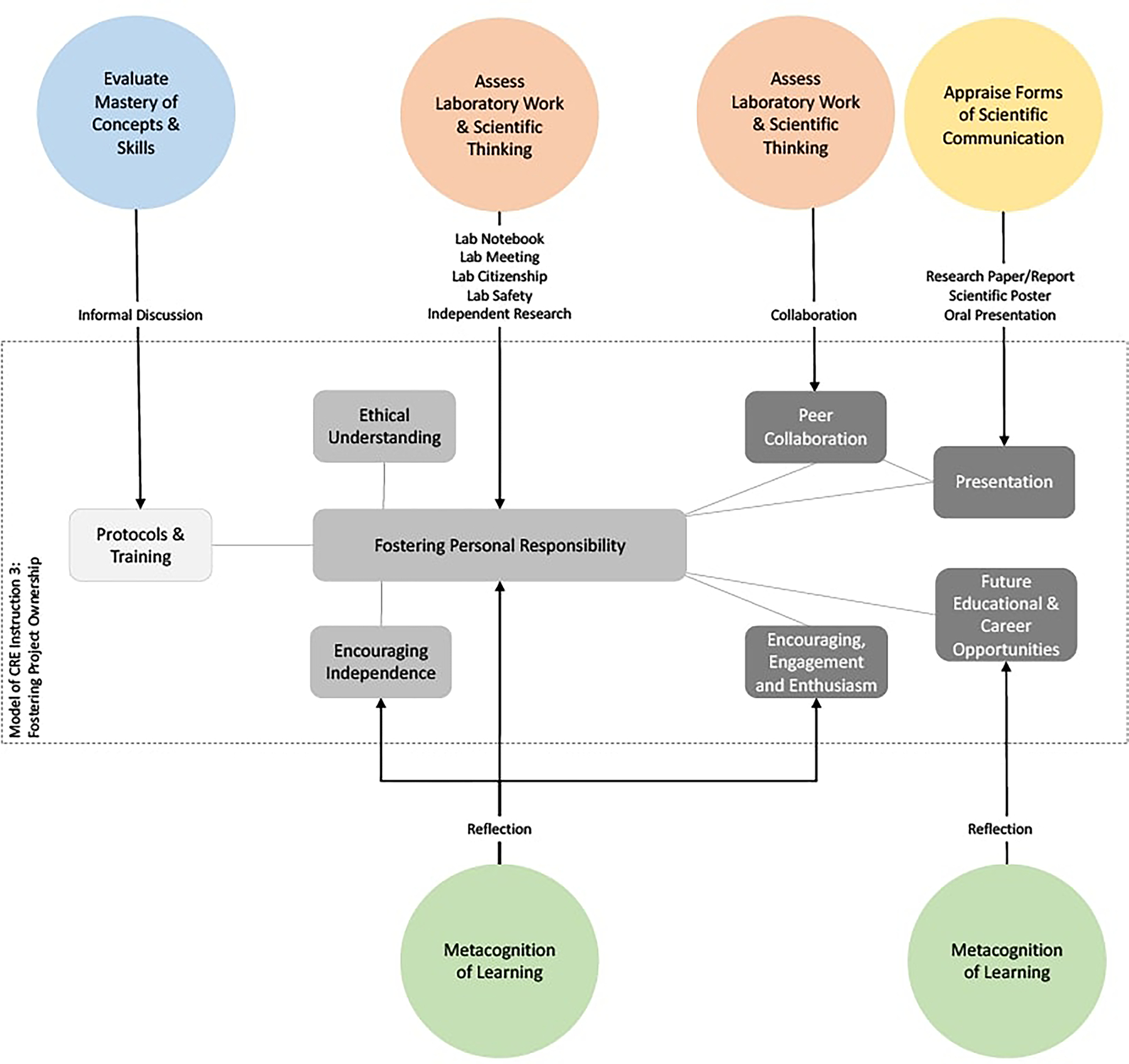
Assessing the Facilitation of Project Ownership: This model has three distinct stages. The first stage relates to development of understanding concerning protocol usage. The second stage relates to the fostering of the student’s sense of personal responsibility. The third stage involves situating the student within the broader scientific context. The model presents the aims and practices of assessment applied at each of these stages.

The educational practice of a CRE involves a desired transition of the student from being a more passive learner of knowledge to being an active producer of knowledge who is integrated into a larger community of researchers. This transition, in which the student has a sense of ownership over their work and responsibility over their research and learning, is an aim of CRE pedagogy and has important ramifications to being a student researcher (Hanauer, et al., 2022). Furthermore, prior research has shown that the development of a sense of project ownership differentiates between an authentic research experience and a more traditional laboratory course. Figure 4 presents the pedagogical and assessment model of fostering project ownership. The model has three stages of development.

- The first stage of fostering project ownership is developing in students a broad understanding and ability to perform a range of scientific protocols. This is because project ownership requires the belief and the ability to actually do science. It is an issue of self-efficacy and mastery of concepts and skills. As such, the first stage of assessment involves evaluating the degree of mastery a student has over a specific protocol. As opposed to prior models, this is enacted here through formative, informal discussions, which also serves to enhance that mastery.
- The second stage of the model aims to develop the student’s sense of personal responsibility. Primary to this process is the promotion and encouragement of the student’s independence. This can involve both emotional supports, the provision of resources, and the allotment of time for the student to ponder the work that they are doing. As reported by faculty, not every question has to be or can be answered immediately. Allowing a student to think about their work and what *they* think should be done is an important aspect of a CRE education. Accordingly, a central component of the assessment model here is having the student reflect on their work. The task of assessment here thus expands beyond the instructor to student as well.
  A different aspect of both fostering and assessing responsibility and ownership over one’s research involves a series of behaviors related to scientific work. Faculty report assessing lab citizenship, collaboration and lab safety protocols. Being responsible includes behaving in appropriate ways in the laboratory and as such these aspects of the students’ work are evaluated. Some faculty also reported that having the student propose projects that extend the ongoing classroom research project allowed them to assess the degree of independence of the student.
- The final stage of the model involves situating the student-researcher within a broader scientific context. Talking with the student about future careers and educational opportunities, and providing encouragement and enthusiasm for the work the student is doing positions the student at the center of their own development. Project ownership involves pride in the research one is doing and seeing ways in which this work can be developed beyond the specific course. Once again, reflection plays a central role in assessing and facilitating this, and occurs as an informal and ongoing process.

In parallel, the outcomes of the research the student does is reported using established modes of scientific communication. A student is responsible for reporting their work using oral presentations, scientific posters, research papers and reports. At this point, they will receive feedback on their work in both formal and informal ways. One important aspect of this reporting is the real-world evaluation of their output. Other peer student researchers may respond, in addition to faculty and scientists beyond the classroom. Having ownership over one’s research also includes an understanding that the work will be evaluated beyond the classroom grade and that the work itself is part of a far larger community of scientists. In this sense, the evaluation of the scientific output facilitates ownership of the research itself.

## DISCUSSION

The main aim of this paper is to explore how assessment of students engaged in course-based research is implemented and aligned with the educational goals of this form of pedagogy. In terms of constructive alignment, the aims of any assessment program should reflect and support defined instructional objectives. Assessment of scientific inquiry, as is typically implemented in traditional labs, focus on mastery of the components of research (see Wenning 2007 for an example). The aim of instruction and assessment within a traditional lab is to make sure that a defined procedure has been mastered by the student so that in some future course or scientific project, the student knows how to perform it. In the traditional lab, grading is evidence of qualification for the student’s ability to function in a future scientific activity. Failure, if it happens, is indeed failure and a reason for not progressing further.

In contrast, a CRE aims to provide the student with an authentic research experience in which they are contributors of research data that is useful for advancing science. As such, mastery is a necessary but not sufficient aim of assessment. As specified by instructors in this study, mastery of concepts, quantitative thinking and skills is important in order to conduct and understand a scientific process; but this is situated in relation to the actual performance of scientific research (also an aim of assessment), which involves an understanding of how to communicate science and ownership over one’s learning and research activity. Thus, from the perspective of what to assess, it is clear that assessment in a CRE needs a broader approach than the assessment program of traditional labs. In this study, four aims of assessment were defined by experienced CRE instructors: 1) Assessing Laboratory Work and Scientific Thinking; 2) Evaluating Mastery of Concepts, Quantitative Thinking and Skills; 3) Appraising Forms of Scientific Communication; and 4) Metacognition of Learning.

The alignment between these assessment aims and the aims of CRE instruction is further explicated here. Across the instructional aims of Facilitating Being a Scientist and Generating Data, Developing Procedural Knowledge, and Fostering Project Ownership, the four aims of assessment were seen to provide ways of collecting useful data that supports the progress of students towards these stated aims of CRE instruction. With regard to how assessment data is collected in a CRE, there are particular relationships between formal and informal assessment and the formative and summative approaches. Summative assessment with formalized tools tended to be at the beginning and end of a research process, in relation to first the development of required mastery of concept and skills and last the evaluation of scientific outputs, which are the products of the research. Mastery can be evaluated using tests and exams, while products can be evaluated using rubrics. In contrast, during the process of conducting the research project, the emphasis is on providing feedback to students to help support the ongoing work. This includes the use of a range of laboratory practices, such as lab notebook documentation and lab meetings. And while assessment data is collected, the response is often informal and formative with the aim of supporting the student to further their research.

Beyond collecting assessment data, there is also a particular way in which assessment, evaluation and grading manifest in a CRE setting. The terms of assessment, evaluation and grading are often used interchangeably. But these terms relate to different concepts. Assessment is primarily a data collection and interpretation task; evaluation is a judgement in relation to the data collected; and grading is a definitive decision expressed as a number or letter as to the final quality of the work of a student. The majority of institutions require grades for a CRE. But not all things that are assessed in a CRE need to be graded. In particular, informal discussion with students of the different aspects of the scientific tasks students are performing allows the instructor to provide supportive feedback that facilitates the scientific inquiry. This informal, formative assessment does not require a grade directly. At the same time, there is a role for assessing and grading the underpinning knowledge, behaviors (such as lab citizenship, attendance, participation, collaboration and lab safety), and scientific outputs of the students. Thus, there is a two-tiered assessment and grading process in which, during the process of scientific inquiry, which is the majority of the course time, assessment data is collected but not graded; however, the knowledge, skills, behaviors and outcomes are graded. Since the aim of the whole course is to give the student the experience of being a researcher and to produce scientific data, providing facilitative feedback based on assessment during the research process helps the student to complete the tasks in a meaningful way. The grading of the underpinning knowledge, skills and behaviors also facilitates the work that is conducted in laboratory. Without appropriate mastery and behavior, the lab research will not be possible. Thus, once again, the form of assessment supports the progress of authentic research. As presented in this study, the way to grade a CRE is to differentiate the framing of the research that is conducted from the process of doing the research; provide extensive formative assessment in an informal manner throughout the research process; grade the underpinning components of knowledge, skill and behavior; and provide a final grade which weights the quality of the work and the output that is produced. The aim should be for every student to be successful in the research process and assessment should facilitate this work.

The assessment and grading practices presented here are clearly facilitative of student learning. First, knowledge, skills and behaviors are measured because they are foundational for students to productively engage in their research. Second, a large part of the assessment work is directly aimed at providing feedback without penalizing a student through grade assignment. There is extensive informal formative assessment that can be seen as a departure from assessment in more traditional labs and which approximates the type of facilitation that characterize mentor-mentee relationships in authentic research settings (e.g. in individual undergraduate research experiences, postbaccalaureate research opportunities, or during postgraduate research). This mentor-mentee relationship can build trust and counter stereotype threat to enhance persistence and learning. Additionally, an assessment program with extensive informal formative assessments leaves fewer instances when a student might be penalized by grading and suffer the negative psychological effects associated with lower grading. Third, the components of CRE assessment address a broad range of skills, beyond just mastery of procedures, that a student needs as a scientist and a learner. In particular, included within the aims of CRE assessment are scientific communication and metacognition. Scientific communication is an important component of being a researcher, while metacognition not only provides information that can be used to evaluate where a student is and how they are thinking about their work, but also positions the student as an evaluator of their own work. In this case, the task of assessment itself directs the students towards better learning and might explain why CREs improve student learning despite the CRE content not always being directly aligned with lecture content (in comparison to traditional lab). We hypothesize that these various aspects of CRE assessment contribute to the positive outcomes observed for students across many demographics and when compared to the traditional lab.

As presented in the introduction, a CRE poses quite specific challenges in terms of assessment and grading. A primary concern relates to the need to maintain a professional shared research project with contributions from instructor and student, while still assessing and grading a student. As presented here this delicate balancing act is facilitated by using assessment and grading thoughtfully and in a coordinated manner. If the instructor is providing extensive feedback that supports the work of the student and grades the aspects of science that are necessary for the student to succeed, the relationship with the student is different from a relationship in which the teacher is just grading a student. The assessment models presented here provide a framework to facilitate the aims of a CRE without undercutting the broader aims of promoting student learning and persistence in science, and can serve to inform assessment and grading practices in STEM, more generally.

## LIMITATIONS

The data and analyses presented in this study emerged from a collective process with a large number of faculty who all implement CREs through the Science Education Alliance (SEA) program by HHMI. Organized as an inclusive Research and Education Community (iREC), faculty in the SEA program are supported by centralized programming to lead the instruction of research projects with a shared research agenda (Hanauer et al., 2017). This does have some ramifications that limit the generalizability of the current results. First, CREs with different research agendas and that require different procedures may change the ratios of formal and informal assessment and what is considered important for grading. Second, while the instructors do work at a wide range of institutions, they also work together in SEA. There is extensive interaction between instructors facilitated by yearly in-person faculty meetings, monthly science and education seminars, and on-line shared resources. This familiarity, interaction and shared course components can lead to a degree of homogeneity in relation to how procedures such as assessment and grading are conducted. As the SEA community facilitated the current data collection and analysis process, it can limit results by not including a much broader set of underlying CRE educational and scientific designs.

## CONCLUSIONS

CREs are increasingly implemented at institutions of higher learning because they offer a strategy to scale-up opportunities for students to engage in authentic research, which is strongly correlated with an increased persistence in science for a wide range of student populations (Russell et al., 2007; Jordan et al., 2014; Hanauer et al., 2017; Hernandez et al., 2018). However, given that CREs situate the research opportunity within the context of a course, it is critically important that the involvement of course grading does not negatively influence students’ belief in their abilities and willingness to persist in STEM (Hatfield, Brown & Topaz, 2022). As seen in the reviews of the multiple instruments developed for the assessment of students in a CRE, the past tendency has been to conceptualize the goals of CRE as a set of skills, competencies, dispositions and abilities to be gained by students for their future engagement in research (Shortlidge & Brownell, 2016; Zelaya, Blumer & Beck, 2022). The assessment of such externalized goals instead of the actual science and scientific process that is at the core of the CRE can lessen the value of the research students are engaged in and contradict their self-perception as researchers.

In contrast, the study presented here models how faculty actively teaching in a large CRE program have integrated assessment into their CRE pedagogy in a way that supports the actual research that is being conducted. In this way, assessment and grading are directly tied to the intended value and aim of a CRE in providing students with an opportunity to engage in research authentically. This is particularly critical because students’ sense of being a scientist is foundational to long-term persistence in the sciences and inappropriate assessment and grading practices could interfere with the positive social and educational values embedded in a CRE (Hanauer et al., 2017). The models of assessment presented here describe how assessment and grading can be conceptualized and implemented in a way that maintains the student’s authentic sense of being a researcher. The approach to assessment described in this paper, which emerged from an extensive interaction with a large community of faculty who actively teach a CRE, describes ways in which assessment can support the educational and social agenda of a CRE. We hope that this study will encourage other researchers working a wider range of CREs to study their own assessment and grading objectives and practices and consider the ways in which assessment can facilitate and not hinder the student’s research experience.

## Figures and Tables

**Table 1:** Instructor Demographic Characteristics (N=105)

Category	*Frequency*	*Percentage*

***Gender***		
Man	19	18.1%
Woman	52	49.5%
Unlisted	1	1%
No Response	33	31.4%

***Ethnicity Identification***		
Asian	4	3.8%
African American	3	2.9%
Hispanic/Latino	3	2.9%
White	61	58.1%
Multiple	1	1%
No Response	33	31.4%

***Rank***		
Adjunct Professor	2	1.9%
Assistant Professor	18	17.1%
Associate Professor	20	19%
Full Professor	17	16.2%
Instructor	13	12.4%
Other	2	1.9%
No Response	33	31.4%

**Years Teaching in the SEA**		
1	12	11.4%
2	14	13.3%
3	13	12.4%
4	12	11.4%
5	4	3.8%
6 +	17	16.2%
No Response	33	31.4%

**Years Teaching Postsecondary Science**		
2	3	2.9%
3	5	4.8%
4	8	7.6%
5	9	8.6%
6+	47	44.8%
No Response	33	31.4%

**Table 2:** Mean and Standard Deviation for Assessment Knowledge Levels (n=72)

Assessment Term	Mean	Std

***PROGRAM AND INSTRUMENT***		

*Assessment Program*	4.15	1.016

*Student Learning Outcomes*	4.89	.358

*Student Competencies*	4.67	.605

*Formative Assessment*	4.53	.903

*Summative Assessment*	4.50	.964

*Portfolio*	4.22	.982

*Assessment Task*	4.27	.878

*Performance Assessment*	4.03	1.000

*Authentic Assessment*	3.24	1.204

*Alternative Assessment*	3.42	1.017

*Problem Solving Questions*	4.79	.555

*Scenario Questions*	4.57	.766

*Rubrics*	4.92	.278

*Analytic Scales*	3.46	1.067

***Grand Mean***	**4.26**	**0.55**

***ASSESSMENT VALIDATION***		

*Assessment Validity*	3.66	1.068

*Item Discrimination*	3.11	1.228

*Assessment Reliability*	3.65	1.103

*Content Validity*	3.25	1.230

*Item Difficulty*	3.91	1.126

*Inter-rater Reliability*	3.10	1.503

*Intra-rater Reliability*	3.01	1.468

*Internal Consistency*	3.01	1.409

***Grand Mean***	**3.34**	**.35**
	

**Table 3:** Faculty Assessment Confidence Levels (n=72)

Confidence Category	Mean	Std.

*I am confident in my ability to define the important components of my course*	4.47	.6

*I am confident in my ability to define my course in terms of student learning outcomes*	4.43	.65

*I am confident in my ability to design formative assessments*	4.08	.92

*I am confident in my ability to evaluate the quality of the assessments that I have designed*	3.88	.75

*I am confident in my ability to analyze the formative assessments that I have designed*	3.72	.89

*I am confident in my ability to analyze the summative assessments that I have designed*	3.81	.97

*I am confident in my ability to provide students with relevant feedback based on the formative assessments that I have designed*	4.10	.86

*I am confident in my ability to explain to specific students the outcomes of their summative assessment performance*	3.93	.99

*I am confident in my ability to report assessment outcomes to administrators*	3.87	.95

*I am confident that my assessments accurately reflect the teaching objectives of my course*	4.11	.74

*Overall, I am confident in my ability to assess my students appropriately*	4.26	.65

*I am satisfied with my current grading procedures*	4.07	.79

***OVERALL***	**4.04**	**.65**
	

**Table 4:** Assessment aims and practices with frequency of mentions and definitions

Aims of Assessment	Practices	Frequency	Practice Definition (Evaluation of…)

***Assess Laboratory Work and Scientific Thinking: (Skills, practices, thoughts patterns and ethics related to laboratory work)***	**Lab Meeting** **Lab Notebook** **Data Card** **Annotation notebook** **Annotation** ^1^ **Lab citizenship** **Collaboration** **Participation, attendance** **Independent research** **Conference Participation** **Lab Safety** **Informal Discussion** *	12 63 9 5 37 10 5 39 9 14 3	Check current status of student research Student’s ability to record their research and to evaluate research status. Document and organize data collection Note keeping of annotation process Annotation of phage genome Student behavior in the lab Student ability to work together with other student researchers Presence and participation of student Check student ability to conduct bioinformatic research Attending a professional scientific convention Aseptic technique and safe behavior Ad hoc on task instructor-student discussion

	**TOTAL**	**203**	**Community Checking Positive Agreement with Categories = 85.95%**

***Evaluate Mastery of Concepts, Quantitative Thinking and Skills***	**Practical Exams**^2^ **Exams and tests** **Quiz**^3^ Lab Notebook* Reflective Writing* Reports* Article Writing* Informal Discussion*	20 22 72	Check students’ mastery of technical skills in related experiments Students understanding of lectures, reading materials and science Students understanding of concepts (including annotation)

	**TOTAL**	**114**	**Community Checking Positive Agreement with Categories = 80.99%**

***Appraise Forms of Scientific Communication***	**Research Paper/Report** **Scientific Poster** **Oral Presentation** **Peer Review** **Journal Club** **Literature Search** **Informal Communication** **Lab Notebooks** **Lab Meetings** **Elevator Speech**	46 44 45 6 16 11	Students’ ability to participate in writing a research paper Presentation and understanding of research Oral-lecture format of research presentation Students’ ability to evaluate each other’s research Shared reading of primary literature Search for relevant scientific scholarship

	**TOTAL**	**168**	**Community Checking Positive Agreement with Categories = 63.64%**

***Metacognition of Learning***	**Reflection** **Exit Ticket** **Grade Discussion** **Informal Discussion**	2 10	Evaluate students understanding and attitudes to learning and research Checklist of activities related to research and learning

	**TOTAL**	**12**	**Community Checking Positive Agreement with Categories = 76.85%**
	

1 Annotation = Annotation (28) + Bioinformatic work (3) + Group Annotation Assignment (6) = 37

2 Practical Exams = Practical Exams (6) + Lab Practical (14) = 20

3 Quiz = Quiz (65) + Question and Answer Assignment (7) = 72

* Added during the community checking process (no frequency data)
